# Snakes

**Published:** 1888-03

**Authors:** 


### SNAKES.

### DEATHS IN INDIA IN 1886, FROM SERPENTS AND WILD ANIMALS.

During the year 1886 the number of persons killed by wild animals and venomous snakes in Hindostan was 24,841. This is an increase of 1,934 over the figures for the previous year.

The fatalities from snake bites rose from 20,142 to 22,134, while wild animals killed 2,707 persons, as compared with 2,765 in 1885. Tigers were responsible for 928 deaths and wolves for 222, these figures showing 90 more deaths from tigers and 26 fewer from wolves as compared with the preceding year. Elephants, leopards, bears, hyenas, wild boars, bisons, wild hogs, jackals, alligators, crocodiles, wild cats, and panthers had also their quota of victims.

The very large proportion of the total deaths from injuries inflicted by wild animals and snakes take place in the lower provinces of Bengal and in the northwestern provinces and Oude. For example, during 1886 no fewer than 18,805 fatal cases of the aggregate of 24,841 for the whole of India occurred in these districts. In Bengal during the year 477 deaths were caused by jackals and 198 by alligators. The total number of cattle killed throughout India by wild animals and snakes during 1886

was 57,541, as compared with 59,029 in the previous year. With regard to the destruction of wild animals, the figures are far from satisfactory. Comparing the year 1886 with 1885, the number of tigers killed fell from 1,855 to 1,464, of leopards from 5,466 to 4,051, and of bears from 1,874 to 1,668 The decline was chiefly in Bengal and Madras, and the reason has been generally attributed to the smallness of the rewards, whereby shikaris are not encouraged to kill the noxious animals.

There was a falling off in the number of snakes killed from 420,044 to 417,596. The decline was chiefly in Bengal, and the cause assigned is again the small amount available for the payment of rewards. The total amount paid in India during 1886 in rewards for the destruction of wild animals and poisonous snakes was Rs, 1,89,006, against Rs. 2,24,126 in the previous year Of the former amount, Rs. 25,360 were paid for the destruction of snakes. The snakes killed in Bombay were more in number than all the other snakes destroyed over the whole of India.—*Times of India.*

